# Longitudinal associations between physical activity and five risk factors of metabolic syndrome in middle-aged adults in Germany

**DOI:** 10.1186/s13098-023-01062-5

**Published:** 2023-04-26

**Authors:** Laura Cleven, Anna Dziuba, Janina Krell-Roesch, Steffen C. E. Schmidt, Klaus Bös, Darko Jekauc, Alexander Woll

**Affiliations:** 1 Institute of Sports and Sports Science, Karlsruhe Institute of Technology, Engler-Bunte-Ring 15, 76131 Karlsruhe, Germany; 2 Institute of Sport Sciences, Department of Sport Psychology, Goethe University Frankfurt, Ginnheimer Landstraße 39, 60487 Frankfurt, Germany

**Keywords:** Abdominal obesity, Hyperlipidemia, Hypertension, Diabetes, Metabolic syndrome, Physical activity, Exercise, Longitudinal study

## Abstract

**Background:**

We examined the longitudinal association between (change in) physical activity (PA) with new onset of five risk factors of metabolic syndrome among 657 middle-aged adults (mean age 44.1 (standard deviation (SD) 8.6) years) who were free of the respective outcome at baseline, in a longitudinal cohort study spanning over 29 years.

**Methods:**

Levels of habitual PA and sports-related PA were assessed by a self-reported questionnaire. Incident elevated waist circumference (WC), elevated triglycerides (TG), reduced high-density lipoprotein cholesterols (HDL), elevated blood pressure (BP), and elevated blood-glucose (BG) were assessed by physicians and by self-reported questionnaires. We calculated Cox proportional hazard ratio regressions and 95% confidence intervals.

**Results:**

Over time, participants developed (cases of incident risk factor; mean (SD) follow-up time) elevated WC (234 cases; 12.3 (8.2) years), elevated TG (292 cases; 11.1 (7.8) years), reduced HDL (139 cases; 12.4 (8.1) years), elevated BP (185 cases; 11.4 (7.5) years), or elevated BG (47 cases; 14.2 (8.5) years). For PA variables at baseline, risk reductions ranging between 37 and 42% for reduced HDL levels were detected. Furthermore, higher levels of PA (≥ 16.6 METh per week) were associated with a 49% elevated risk for incident elevated BP. Participants who increased PA levels over time, had risk reductions ranging between 38 and 57% for elevated WC, elevated TG and reduced HDL. Participants with stable high amounts of PA from baseline to follow-up had risk reductions ranging between 45 and 87% for incident reduced HDL and elevated BG.

**Conclusions:**

PA at baseline, starting PA engagement, maintaining and increasing PA level over time are associated with favorable metabolic health outcomes.

## Background

Elevated waist circumference (WC), elevated triglycerides (TG), reduced high-density lipoprotein cholesterols (HDL), elevated blood pressure (BP), and elevated blood-glucose (BG) are considered risk factors of metabolic syndrome (MetS) [1]. The prevalence of MetS, and individual risk factors of MetS has increased over the past decades [2]. It is estimated that the prevalence of MetS worldwide ranges between 20 and 25% in the adult population, and up to 80% of diabetes patients are affected [2–4]. These numbers are alarming, particularly considering the economic impact of MetS on health care systems [2, 5]. Therefore, it is critical to identify potential lifestyle-related factors that may decrease the risk of new onset of MetS.

Physical activity (PA) is known as a lifestyle-related factor to impact the metabolic system in general. On a physiological level, PA has an impact on cardiovascular (e.g., improvement in oxygen uptake and transport capacity; reduction of heart rate; increase in stroke volume), hemodynamic (e.g., improvement in blood flow; increase in blood clotting readiness), metabolic (e.g., increase in mitochondria volume; improvement of enzyme activity of the musculature; change of cholesterol composition by improvement of HDL-LDL ratio), and endocrinological systems (e.g., increase in catecholamines, cortisol, growth hormones), amongst others [6–8]. Furthermore, on an endocrinological level, an increase in the exercise-induced hormone Irisin by the skeletal muscle, may cause an increase in energy expenditure by transforming white into brown fat cells (brown-in-white or brite cells), which is linked to an improvement in glucose homeostasis [6, 9]. Due to the direct impact on bodily functions, PA has a positive impact on an individual’s health, and may thus also have a favorable impact on the risk of new onset of individual MetS risk factors.

The World Health Organization (WHO) recommends engaging in at least 150 min of moderate to vigorous PA, or 75 min of vigorous-intensity PA per week, or an equivalent combination of moderate- and vigorous-intensity activity to achieve health benefits [10], which is equivalent to at least 8.3–16.6 metabolic equivalent-hours (METh) per week (respectively 500–1000 MET-minutes per week) [11].

Indeed, a growing body of research has reported a beneficial effect of PA on cardiometabolic risk factors, e.g., being physically active may reduce body weight and BP, elevate HDL, lower TG, and improve insulin resistance [12–17]. Studies have also shown that engaging in higher levels of PA is associated with a reduced risk for incident MetS [18–21]. As it is important to focus on MetS itself, contribution of PA to the risk of new onset of the respective individual risk factors should be considered as well. Longitudinal studies reporting the association between variables of PA and individual risk factors of MetS, combined within one analysis or dataset, are scarce. However, to date, studies focusing on the longitudinal associations between various PA measures with individual factors of MetS in large, population-based samples are missing.

Therefore, the aim of this study was to examine the associations between various PA variables (i.e., habitual and sports-related PA, and change in PA behavior over time) and new onset of five different risk factors of MetS among middle-aged males and females from a community-based sample in South-Western Germany over a period of 29 years.

## Methods

For the reporting of observational studies within this manuscript the STROBE (Strengthening The Reporting of OBservational Studies in Epidemiology) Checklist was adhered.

### Study design and population

The research was conducted in the setting of the ‘Gesundheit zum Mitmachen’ study, which is an ongoing, community-based longitudinal cohort study of middle-aged and older adults living in the city of Bad Schönborn in South-Western Germany with six measurement points (1992, 1997, 2002, 2010, 2015, 2021). Details about the design and methods of the study have been described elsewhere [22, 23]. The study was approved by the ethics committee of the Karlsruhe Institute of Technology (KIT), Germany, and was conducted in accordance with the Declaration of Helsinki. All methods were performed in accordance with pertinent guidelines and regulations. All data was analysed anonymously.

Briefly, individuals residing in Bad Schönborn, Germany from five age strata (i.e., 35, 40, 45, 50, and 55 ± 2 years) were randomly selected from the local residents’ registration offices and were invited to participate in the study in 1992 (initial sample). The response rate of the initial sample was 56%. A non-responder telephone interview showed no significant differences in socioeconomic status (SES), physical health status, and PA between participants and invited non-participants except for migration background [23]. Persons who participated in the study at least once were re-invited for every measurement point, and new samples of participants aged 33–37 years were included at each measurement point to prevent sample attrition. Participation in the study was voluntary and all participants provided written informed consent. At each of the six measurements points, the data assessment took place in Mai and June. Participants provided information about self-rated health status, habitual and sports-related PA, and sociodemographic information through a self-reported questionnaire. All participants also underwent an objective health status examination conducted by a licensed physician, and had a blood sample drawn.

**Fig. 1:**
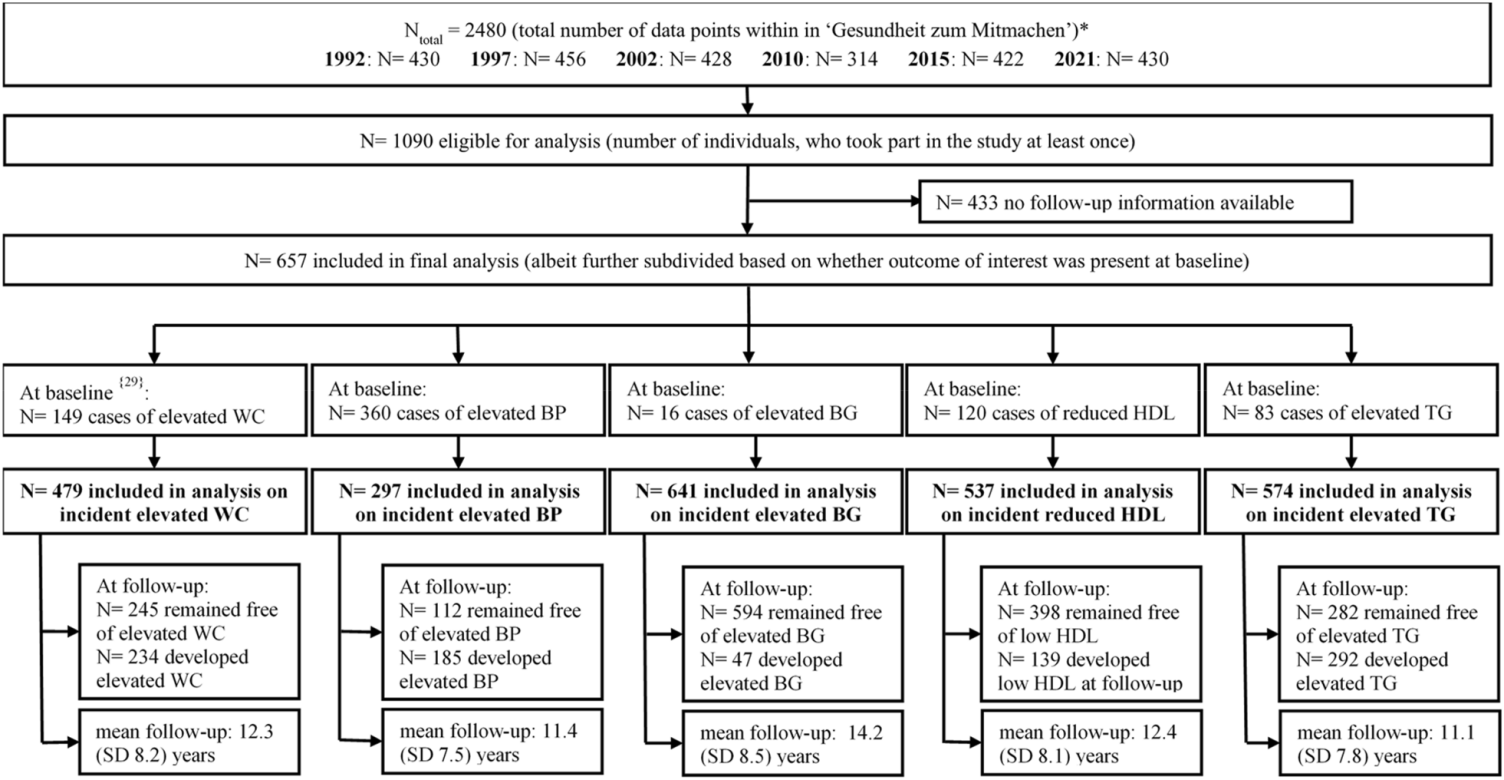
Flow chart of study participation. *N* number of participants, *WC* waist circumference, *BP* blood pressure, *BG* blood-glucose, *HDL* high-density lipoprotein cholesterols, *TG* triglycerides; ^{}^ indicates numbers of cases with missing information. *Persons who participated in the study at least once were re-invited for every measurement point, and new participants were included at each measurement point to prevent sample attrition

For the current analysis, from a total of 1090 individuals who participated in this study, we excluded participants with no longitudinal data information available (N = 433). A total of 657 participants were thus included in the statistical analysis. In a next step, for each of the five outcomes of interest, we excluded individuals with pre-existing risk factor at baseline for the respective analyses (please refer to Fig. 1 for a flow chart of study participation).

### Physical activity assessment (predictor variables)

All information regarding habitual and sports-related PA at baseline examination and follow-up was assessed by a self-reported questionnaire (test–retest reliability after two weeks: *r* > 0.90, α = 0.94) [24].

#### Habitual PA

We calculated an index to quantify the amount of habitual PA at baseline (minutes per week) as indicator for recreational PA level (thus deliberately excluding sports activity participation) by creating a sum index that was derived from participants‘ self-reported information about daily minutes of walking, biking for transportation, as well as working in the household and gardening. For statistical analysis, the sample was divided into three groups (i.e., low: < 75 min per week, medium: 75–149 min per week, and high: ≥ 150 min/ week) based on the calculated index.

#### Sports-related PA

An index for the amount of sports-related PA at baseline was calculated from information provided by participants about frequency (i.e., number of weekly exercise sessions), duration (i.e., minutes per session), intensity (i.e., not very intense, moderately intense with some sweating, and highly intense with much sweating), and type of weekly sports-related PA (i.e., structured/ non structured; organized/ non-organized; e.g. playing soccer, tennis, running, hiking etc.) [25]. For statistical analyses, the sample was divided into three groups (i.e., low: < 75 min per week, medium: 75–149 min per week, high: ≥ 150 min per week) to examine the influence of the cut-off points based on the global recommendation on PA level [26]. Furthermore, to consider the intensity level of the activity, every type of sport was assigned a specific MET-value [27], and by multiplication with the time spent carrying out the respective activity, sports-related PA level in METh per week at baseline was calculated. The global PA recommendation is equivalent to at least 8.3–16.6 METh per week [11]. Thus, for statistical analysis, the sample was divided into three groups (not active: < 8.3 METh per week, moderately active: 8.3–16.5 METh per week, highly active: ≥ 16.6 METh/week) based on the sports-related PA level to examine the influence of thresholds based on the global recommendation.

#### Change of sports-related PA

To assess the change in minutes of sports-related PA per week, differences in PA level between baseline and the latest follow-up examination were calculated individually for each participant. Four categories by applying a 150 min per week threshold, based on WHO guidelines on physical activity and sedentary behavior [10] were calculated. The four categories were *stable inactive* (i.e., individuals who continuously reported less than 150 min per week sports-related PA); *quits activity* (i.e., participants who reported more than 150 min per week sports-related PA at baseline but not follow-up); *starts activity* (i.e., individuals who reported less than 150 min per week sports-related PA at baseline but more than 150 min per week at follow-up); and *stable active* (i.e., participants who reported continuously more than 150 min per week of sports-related PA). Similarly, to assess the change in METh of sports-related PA, differences in METh level at baseline examination compared to the latest follow-up were calculated. By applying the threshold for METh recommended by the U.S. Department of Health and Human Services [11], four groups were created: *Stable low* (i.e., individuals who continuously reported less than 16.6 METh per week sports-related PA), *decreasing* (i.e., participants who reported more than 16.6 METh per week sports-related PA at baseline but not at follow-up); *increasing* (i.e., individuals who reported less than 16.6 METh per week sports-related PA at baseline but more than 16.6 METh per week at follow-up); and *stable high* (i.e., participants who reported continuously more than 16.6 METh per week sports-related PA).

### Individual factors of metabolic syndrome assessment (outcomes of interest)

At baseline and each follow-up examination, the status of risk factors of MetS was assessed through examination by a licensed physician and corroborated by self-reported information on medication intake in the questionnaire. We defined having or developing new onset of risk factors of MetS, when the respective risk factors (i.e., elevated WC, elevated TG, reduced HDL, elevated BP, or elevated BG) were present at baseline examination or at follow-up [1].

#### Elevated waist circumference

WC (in cm) was assessed at baseline and at each follow-up examination by standardized measurement. A participant was classified as having elevated WC when the WC was greater than 102 cm for males, and greater than 88 cm for females, respectively [28, 29].

#### Blood lipids (triglycerides and high-density lipoprotein cholesterol)

Blood lipids were assessed at baseline and at each follow-up examination by standardized measurement. Participants were classified as having elevated TG when the levels were greater than 150 mg/dL, and as having reduced HDL when the levels were lower than 40 mg/dL for males and lower than 50 mg/dL for females, respectively [1]. Furthermore, a participant was classified as having critical level of blood lipids (i.e., elevated TG or reduced HDL) if the diagnosis had been made previously by a physician, or if the participant was on medication for the respective condition, i.e., participants were asked: ‘Do you take any medication to lower blood lipids?’.

#### Elevated blood pressure

BP was assessed at baseline and each follow-up examination by standardized measurement. A participant was classified as having elevated BP if the systolic value was greater than 130 mmHg, or if the diastolic value was greater than 85 mmHg. Furthermore, a participant was classified as having elevated BP if hypertension diagnosis had been made previously by a physician, or if the participant was on BP medication [1], i.e., participants were asked: ‘Do you take any medication to lower your blood pressure?’.

#### Elevated blood-glucose level

BG was determined by a physician at baseline and each follow-up examination based on BG levels (i.e., fasting BG level ≥ 100 mg/dL [1], non-fasting BG level ≥ 200 mg/dL [30] for measurement points 1992 to 2015; and HbA1c ≥ 6,5% in 2021 [30]). Furthermore, a participant was classified as having elevated BG level, if a diabetes diagnosis had been made previously by a physician or if the participant was on medication, i.e., participants were asked: ‘Do you take any medication to lower blood-glucose levels?’.

#### Assessment of confounders

Traditional demographic variables (e.g., age and sex) were assessed though self-reported questionnaire. We also determined socio-economic status (SES) based on information provided by participants about formal education and professional status of themselves or their significant others (usually the spouse), if participants were not working. Four SES categories were used for statistical analysis, i.e., low, mid/ low, mid/ high and high SES [23, 31]. Furthermore, comorbidities at study entry related to the risk factors of MetS (i.e., elevated WC, elevated TG, reduced HDL, elevated BP, and elevated BG, respectively) were determined.

### Statistical analysis

Selected sociodemographic, behavioral, and health-related characteristics of the participants were used to characterize the cohort at baseline by using means (M) and standard deviation (SD). Cox proportional hazards regression models were calculated to examine the associations between various PA predictor variables (i.e., habitual and sports-related PA at baseline, and change in sports-related PA level from baseline to follow-up; variables were nominal coded or dummy coded), and the risk of new onset of a MetS risk factor. Only participants who were free of the respective outcome of interest at baseline, and had at least one follow-up measurement were included in the analysis. Date of study entry was used as baseline, and was determined individually for each participant. Follow-up time (in months) was calculated individually for each participant from baseline until the first event (i.e., incident elevated WC, elevated TG, reduced HDL, elevated BP, elevated BG) or loss to follow-up, whichever happened first. Missing values were treated as missing at random, which yielded in a pairwise deletion of cases with at least one missing value among either predictor or outcome variable for each analysis. Numbers of missing values are provided in the result table for each category of PA, respectively. We calculated Hazard Ratios (HR) and 95% confidence intervals (CI) based on regression analyses for each category of PA as compared to the least active group which was always set as reference group (we also indicated the reference group in the results tables). For each predictor, we ran two sets of models: models adjusted for traditional confounding variables, i.e., age, sex and SES (Model 1), and models additionally adjusted for comorbidities at study entry related to the risk factors of MetS (i.e., elevated WC, reduced HDL, elevated TG, elevated BP, elevated BG) respectively. This means that, for example, in the models where the outcome of interest was elevated WC, we additionally adjusted for status of HDL, TG, BP and BG at study entry. However, it is recommended to do one adjustment per every 15 cases of incident event (per incident outcome of interest). Therefore, in order to avoid over-adjustment, we only report Model 1 for the outcome of interest blood-glucose, as we only had 47 participants who developed incident elevated blood-glucose levels during time under observation. Analyses were performed using SPSS software version 27, and using the conventional alpha level of 0.05 to determine statistical significance. A HR > 1.0 was considered as indicating an elevated risk for incident risk factors of MetS, a HR < 1.0 was considered as indicating a reduced risk for incident risk factors of MetS.

## Results

### Sample characteristics

Data were available on a total of 657 individuals with at least two study visits (i.e., baseline and at least one follow-up). Mean age at baseline was 44.1 (SD 8.6; range: 30–74) years, and 52.1% of participants were female. Habitual PA was performed for a mean of 349.4 (SD 274.0) minutes per week. Sports-related PA was performed for a mean of 96.3 (SD 128.4) minutes per week, and with a mean of 11.6 (SD 15.5) METh per week, and it was most frequently performed for less than 75 min per week (53.3%). Sample size and demographics differed for the statistical analyses depending on the outcome of interest. Detailed information about baseline demographics of each study sample for the five different outcomes of interest (i.e., elevated WC, elevated TG, reduced HDL, elevated BP, elevated BG) are shown in Table 1.

**Table 1:** Demographics of study participants (stratified by sex) at baseline for each outcome of interest

	Outcomes of interest									
	Elevated WC (N = 479)		Elevated TG (N = 574)		Reduced HDL (N = 537)		Elevated BP (N = 297)		Elevated BG (N = 641)	
	Male	Female	Male	Female	Male	Female	Male	Female	Male	Female
Elevated WC, N (%)	–	–	50 (19.5)^{5}^	60 (18.9)^{40}^	48 (18.5)^{2}^	46 (16.5)^{36}^	10 (9.4)^{2}^	29 (15.2)^{31}^	62 (20.4)^{2}^	67 (19.9)^{42}^
Elevated TG, N (%)	44 (18.3)	18 (7.5)	–	–	34 (13.1)	12 (4.3)	21 (19.8)	10 (5.2)	58 (19.1)	24 (7.1)
Reduced HDL, N (%)	39 (16.3)	37 (15.5)	31 (12.1)	52 (16.4)	–	–	20 (18.9)	35 (18.3)	54 (17.8)	63 (18.7)
Elevated BP, N (%)	149 (62.1)	106 (44.4)	171 (66.8)	137 (43.1)	173 (66.8)	122 (43.9)	–	–	199 (65.5)	148 (43.9)
Elevated BG, N (%)	6 (2.5)	2 (0.8)	10 (3.9)	5 (1.6)	9 (3.5)	4 (1.4)	1 (0.9)	2 (1.0)	-	-
Confounders										
N	240	239	256	318	259	278	106	191	304	337
Age [years], mean (SD)	43.3 (8.4)	44.0 (8.9)	44.5 (8.5)	44.1 (8.5)	44.4 (8.7)	44.1 (8.5)	41.0 (8.3)	41.9 (8.2)	44.3 (8.5)	43.8 (8.5)
SES, mean (SD)	3.2 (0.9)^{2}^	2.9 (0.9)^{5}^	3.1 (0.9)^{4}^	2.8 (0.9)^{7}^	3.1 (0.9)^{4}^	2.8 (0.9)^{5}^	3.2 (0.9)^{1}^	2.9 (0.9)^{6}^	3.1 (0.9)^{3}^	2.8 (0.9)^{8}^
Low (%)	4.6	10.0	5.1	10.7	5.4	10.1	2.8	7.9	5.9	10.1
Low/medium (%)	23.3	16.3	25.0	19.5	23.9	19.4	27.4	21.5	23.7	19.0
Medium/high (%)	24.2	44.4	24.6	43.4	26.6	44.6	19.8	41.4	25.3	43.6
High (%)	47.1	27.2	43.8	24.2	42.5	24.1	49.1	26.2	44.1	24.9
Predictors										
Habitual PA [min/week], mean (SD)	376.1 (302.9)	307.5 (239.1)	375.8 (305.5)	317.8 (233.9)	388.3 (313.8)	324.1 (237.9)	366.8 (288.0)	296.7 (209.2)	379.0 (310.5)	319.6 (230.1)
Low (< 75) (%)	4.6	5.9	6.3	5.3	5.4	5.4	1.9	6.3	5.3	5.0
Medium (75–149) (%)	22.5	26.8	21.1	23.9	22.4	24.1	23.6	25.7	21.7	22.8
High (≥ 150) (%)	72.9	67.4	72.7	70.8	72.2	70.5	74.5	68.1	73.0	72.2
Sports-related PA [min/week], mean (SD)	116.0 (148.4)	104.7 (126.8)	105.3 (144.4)	90.4 (119.2)	105.5 (142.6)	98.1 (125.8)	79.2 (93.8)	92.9 (121.5)	102.9 (137.9)	90.7 (120.1)
Inactive (< 75) (%)	47.1	47.7	50.4	55.0	51.0	53.2	56.6	53.4	52.0	55.5
Medium (75–149) (%)	26.3	25.1	25.8	20.8	25.1	20.1	26.4	23.0	24.0	20.5
High (≥ 150) (%)	26.7	27.2	23.8	24.2	23.9	26.6	17.0	23.6	24.0	24.0
Sports-related PA [METh/week], mean (SD)	14.1 (17.9)	12.3 (15.2)^ {1}^	12.4 (17.4)	10.9 (14.6)^{1}^	12.9 (17.3)	12.0 (15.3)^ {1}^	9.5 (11.4)	10.5 (14.0) ^{1}^	12.4 (16.7)	10.9 (14.5) ^{1}^
Low (< 8.3) (%)	46.3	51.9	51.6	57.9	51.0	55.0	55.7	57.6	51.3	57.3
Medium (8.3–16.5) (%)	25.4	22.6	24.6	17.6	23.9	18.0	25.5	18.8	24.3	18.7
High (≥ 16.6) (%)	28.3	25.1	23.8	24.2	25.1	26.6	18.9	23.0	24.3	23.7

*N* sample size, *SD* standard deviation, *kg* kilograms, *m* meters, *cm* centimeters, min minutes, *PA* physical activity, *METh* metabolic equivalent hours; *WC* waist circumference, *BP* blood pressure, *BG* blood-glucose, *HDL* high-density lipoprotein cholesterols, *TG* triglycerides, ^{}^ indicates number of N missing. Elevated waist circumference: ≥ 102 cm for males, ≥ 88 cm for females; elevated triglycerides: ≥ 150 mg/dL and/or specific medication; reduced HDL-cholesterols: < 40 mg/dL for males, < 50 mg/dL for females and/or specific medication; elevated blood pressure: systolic value ≥ 130 mmHg or diastolic value ≥ 85 mmHg and/or specific medication; elevated blood glucose: fasting blood-glucose level ≥ 100 mg/dL or non-fasting blood-glucose level ≥ 200 mg/dL or HbA1c ≥ 6,5% and/or specific medication

### Association of PA with new onset of elevated waist circumference

Of all participants included in the analysis, 224 (46.8%) had one follow-up assessment, 101 (21.1%) participants had two follow-up assessments, 74 (15.4%) participants had three follow-up assessments, 49 (10.2%) had four follow-up assessments, and 31 (6.5%) participants had 5 follow-up assessments. The mean (SD) times of follow-up assessments was of 2.1 (1.3). After a mean follow-up of 12.3 (SD 8.2, range 5–29) years, 234 (48.9%) participants developed incident elevated WC (please refer to Fig. 1) with 5872 person-years (sum of the time spans for each participant under observation).

Habitual and sports-related PA at baseline were not associated with the risk of incident elevated WC (please refer to Table 2).

**Table 2:** Association between PA variables and the risk of incident elevated waist circumference

No. with incident elevated waist circumference (N = 234, 48.9%)						
	No. at risk (N = 479)	No. incident elevated waist circumference (% of No. at risk)	Model 1 adjusted Hazard Ratio (95% CI)	p-value	Model 2 adjusted Hazard Ratio (95% CI)	p-value
Habitual PA level at baseline [min/week]						
Low (< 75)	25	14 (2.9)	ref		ref	
Medium (75–149)	118	58 (12.1)	0.77 (0.42–1.41)	0.397	0.78 (0.42–1.43)	0.417
High (≥ 150)	336	162 (33.8)	0.73 (0.41–1.30)	0.284	0.72 (0.40–1.27)	0.253
Sports-related PA level at baseline [min/week]						
Low (< 75)	227	109 (22.8)	ref		ref	
Medium (75–149)	123	66 (13.8)	1.05 (0.77–1.44)	0.749	1.08 (0.79–1.49)	0.618
High (≥ 150)	129	59 (12.3)	0.92 (0.67–1.26)	0.594	0.88 (0.64–1.21)	0.424
Sports-related intensity level at baseline [METh/week] ^{1}^						
Not intensively active (< 8.3)	235	110 (23.0)	ref		ref	
Moderately intensively active (8.3–16.5)	115	59 (12.3)	1.10 (0.80–1.52)	0.552	1.07 (0.78–1.48)	0.669
Highly intensively active (≥ 16.6)	128	65 (13.6)	1.09 (0.80–1.48)	0.593	1.06 (0.77–1.44)	0.725
Change in sports-related PA over time [min/week] ^{19}^						
Stable inactive (< 150– < 150)	234	115 (24.0)	ref		ref	
Quits activity (≥ – < 150)	51	27 (5.6)	0.96 (0.63–1.47)	0.859	0.91 (0.59–1.39)	0.656
Starts activity (< 150– ≥ 150)	111	58 (12.1)	0.75 (0.54–1.04)	0.084	0.78 (0.56–1.07)	0.123
Stable active (≥ 150– ≥ 150)	64	26 (5.4)	0.69 (0.45–1.05)	0.084	0.66 (0.43–1.01)	0.057
Change in sports-related intensity level over time [METh/week] ^{4}^						
Stable low (< 16.6– < 16.6)	250	126 (26.3)	ref		ref	
Decreasing (≥ 16.6– < 16.6)	47	29 (6.1)	1.32 (0.87–1.98)	0.188	1.37 (0.91–2.07)	0.130
Increasing (< 16.6– ≥ 16.6)	98	42 (8.8)	**0.61 (0.43–0.88)**	0.007*	**0.62 (0.45–0.92)**	**0.015**
Stable high (≥ 16.6– ≥ 16.6)	80	35 (7.3)	0.72 (0.49–1.05)	0.084	0.70 (0.48–1.02)	0.060

*CI* confidence interval, *METh* metabolic equivalent hours, *min* minutes, *N* number of participants, *No*. Number, *PA* physical activity, *ref* reference group, *SES* socio-economic status, *WC* waist circumference. Model 1: adjusted for age, sex and SES; Model 2: adjusted for age, sex, SES, and comorbidities with regard to MetS risk factors at study entry. ^{}^ indicates number of N missing. Significant values (p < 0.05) are in [bold]; significant values after Bonferroni correction for multiple testing are indicated by [*]

Increasing sports-related PA from < 16.6 METh per week at baseline to ≥ 16.6 METh per week at follow-up, was associated with a decreased risk of incident elevated WC (HR 0.62, 95% CI 0.45–0.92, p = 0.015), compared to participants with a stable low sports-related PA level (Table 2).

### Association of PA with incident elevated triglycerides

Of all participants included in the analysis, 242 (42.2%) had one follow-up assessment, 133 (23.2%) participants had two follow-up assessments, 95 (16.6%) participants had three follow-up assessments, 64 (11.1%) had four follow-up assessments, and 40 (7.0%) participants had 5 follow-up assessments. The mean (SD) times of follow-up assessments was of 2.2 (1.3). After a mean follow-up of 11.1 (SD 7.8, range 5–29) years, 292 (50.9%) participants developed incident elevated TG (please refer to Fig. 1) with 6360 person-years (sum of the time spans for each participant under observation).

Habitual and sports-related PA at baseline was not associated with the risk of incident elevated TG (please refer to Table 3).

**Table 3:** Association between PA variables and the risk of incident elevated triglycerides

No. with incident elevated triglycerides (N = 292, 50.9%)						
	No. at risk (N = 574)	No. incident elevated triglycerides (% of No. at risk)	Model 1 adjusted Hazard Ratio (95% CI)	p-value	Model 2 adjusted Hazard Ratio (95% CI)	p-value
Habitual PA level at baseline [min/week]						
Low (< 75)	33	19 (3.3)	ref		ref	
Medium (75–149)	130	58 (10.1)	0.98 (0.57–1.69)	0.947	1.24 (0.68–2.26)	0.489
High (≥ 150)	411	215 (37.5)	1.14 (0.70–1.88)	0.601	1.36 (0.78–2.35)	0.277
Sports-related PA level at baseline [min/week]						
Low (< 75)	304	162 (28.2)	ref		ref	
Medium (75–149)	132	68 (11.8)	0.92 (0.69–1.24)	0.596	1.04 (0.77–1.42)	0.793
High (≥ 150)	138	62 (10.8)	0.87 (0.65–1.17)	0.369	1.02 (0.74–1.40)	0.912
Sports-related intensity level at baseline [METh/week] ^{1}^						
Not intensively active (< 8.3)	316	170 (29.6)	ref		ref	
Moderately intensively active (8.3–16.5)	119	62 (10.8)	0.97 (0.72–1.30)	0.818	1.06 (0.78–1.44)	0.733
Highly intensively active (≥ 16.6)	138	60 (10.5)	0.83 (0.62–1.12)	0.224	0.96 (0.70–1.33)	0.814
Change in sports-related PA over time [min/week] ^{28}^						
Stable inactive (< 150– < 150)	296	167 (29.1)	ref		ref	
Quits activity (≥ 150– < 150)	52	29 (5.1)	0.95 (0.64–1.41)	0.800	1.08 (0.72–1.63)	0.705
Starts activity (< 150– ≥ 150)	119	50 (8.7)	**0.62 (0.45–0.85)**	0.003*	**0.62 (0.44–0.88)**	0.007*
Stable active (≥ 150– ≥ 150)	79	36 (6.3)	0.76 (0.53–1.10)	0.141	0.87 (0.59–1.29)	0.484
Change in sports-related intensity level over time [METh/week] ^{13}^						
Stable low (< 16.6– < 16.6)	318	181 (31.5)	ref		ref	
Decreasing (≥ 16.6– < 16.6)	58	29 (5.1)	0.88 (0.59–1.31)	0.530	1.05 (0.69–1.59)	0.823
Increasing (< 16.6– ≥ 16.6)	110	45 (7.8)	**0.58 (0.42–0.81)**	0.001*	**0.63 (0.44–0.89)**	0.009*
Stable high (≥ 16.6– ≥ 16.6)	75	30 (5.2)	**0.66 (0.45–0.98)**	**0.037**	0.76 (0.50–1.14)	0.182

*CI* confidence interval, *METh* metabolic equivalent hours, *min* minutes, *N* number of participants, *No.* Number, *PA* physical activity, ref reference group, *SES* socio-economic status, *TG* triglycerides. Model 1: adjusted for age, sex and SES; Model 2: adjusted for age, sex, SES, and comorbidities with regard to MetS risk factors at study entry. ^{}^ indicates number of N missing. Significant values (p < 0.05) are in [bold]; significant values after Bonferroni correction for multiple testing are indicated by [*]

Participants who reported starting sports-related PA from < 150 min per week at baseline to ≥ 150 min per week at follow-up, had a decreased risk of incident elevated TG (HR 0.62, 95% CI 0.44–0.88, p = 0.007) as compared to participants who remained continuously inactive. In addition, an increase in sports-related PA from < 16.6 METh per week at baseline to ≥ 16.6 METh per week at follow-up, was associated with a decreased risk of incident elevated TG (HR 0.63, 95% CI 0.44–0.89, p = 0.009), compared to participants with a stable low sports-related PA level (Table 3).

### Association of PA with incident reduced high-density lipoprotein cholesterols

Of all participants included in the analysis, 248 (46.2%) had one follow-up assessment, 108 (20.1%) participants had two follow-up assessments, 88 (16.4%) participants had three follow-up assessments, 54 (10.1%) had four follow-up assessments, and 39 (7.3%) participants had 5 follow-up assessments. The mean (SD) times of follow-up assessments was of 2.1 (1.3). After a mean follow-up of 12.4 (SD 8.1, range 5–29) years, 139 (25.9%) participants developed incident reduced HDL (please refer to Fig. 1) with 6661 person-years (sum of the time spans for each participant under observation).

Habitual PA was not associated with the risk of incident reduced HDL. Engaging in sports-related PA between 75 and 149 min per week at baseline was associated with a decreased risk of incident reduced HDL (HR 0.59, 95% CI 0.37–0.95, p = 0.031), and engaging in high sports-related PA level (≥ 150 min per week) was also associated with a decreased risk of incident reduced HDL (HR 0.63, 95% CI 0.40–0.99, p = 0.048), compared to sports-related PA carried out for less than 75 min per week (please refer to Table 4). With regard to the METh of sports-related PA, being physically active with more than 16.6 METh per week at baseline was associated with a decreased risk of incident reduced HDL (HR 0.58, 95% CI 0.36–0.93, p = 0.024), compared to participants who were not active (Table 4).

**Table 4:** Association between PA variables and the risk of incident reduced high-density lipoprotein cholesterols

No. with incident reduced HDL (N = 139, 25.9%)						
	No. at risk (N = 537)	No. incident reduced HDL (% of No. at risk)	Model 1 adjusted Hazard Ratio (95% CI)	p-value	Model 2 adjusted Hazard Ratio (95% CI)	p-value
Habitual PA level at baseline [min/week]						
Low (< 75)	29	11 (1.9)	ref		ref	
Medium (75–149)	125	31 (5.4)	0.85 (0.41–1.74)	0.648	0.86 (0.41–1.79)	0.683
High (≥ 150)	383	97 (16.9)	0.77 (0.40–1.49)	0.442	0.72 (0.37–1.40)	0.332
Sports-related PA level at baseline [min/week]						
Low (< 75)	280	87 (15.2)	ref		ref	
Medium (75–149)	121	26 (4.5)	**0.61 (0.39–0.96)**	**0.034**	**0.59 (0.37–0.95)**	**0.031**
High (≥ 150)	136	26 (4.5)	**0.63 (0.41–0.98)**	**0.041**	**0.63 (0.40–0.99)**	**0.048**
Sports-related intensity level at baseline [METh/week] ^{1}^						
Not intensively active (< 8.3)	285	88 (15.4)	ref		ref	
Moderately intensively active (8.3–16.5)	112	26 (4.5)	0.72 (0.46–1.13)	0.149	0.68 (0.43–1.09)	0.107
Highly intensively active (≥ 16.6)	139	25 (4.4)	**0.59 (0.37–0.93)**	**0.022**	**0.58 (0.36–0.93)**	**0.024**
Change in sports-related PA over time [min/week] ^{24}^						
Stable inactive (< 150– < 150)	276	86 (15.0)	ref		ref	
Quits activity (≥ 150– < 150)	38	8 (1.4)	0.61 (0.30–1.27)	0.189	0.66 (0.32–1.38)	0.269
Starts activity (< 150– ≥ 150)	120	23 (4.0)	**0.54 (0.34–0.87)**	**0.011**	**0.43 (0.25–0.74)**	0.002*
Stable active (≥ 150– ≥ 150)	79	18 (3.1)	0.74 (0.44–1.23)	0.245	0.67 (0.39–1.16)	0.156
Change in sports-related intensity level over time [METh/week] ^{11}^						
Stable low (< 16.6– < 16.6)	86	92 (16.1)	ref		ref	
Decreasing (≥ 16.6– < 16.6)	101	9 (1.6)	0.56 (0.27–1.15)	0.114	0.62 (0.30–1.28)	0.193
Increasing (< 16.6– ≥ 16.6)	50	17 (3.0)	**0.48 (0.28–0.81)**	0.006*	**0.43 (0.24–0.77)**	0.004*
Stable high (≥ 16.6– ≥ 16.6)	289	16 (2.8)	**0.57 (0.33–0.98)**	**0.040**	**0.55 (0.31–0.97)**	**0.040**

*CI* confidence interval, *METh* metabolic equivalent hours, *HDL* high-density lipoprotein cholesterols, *min* minutes, *N* number of participants, *No.* Number, *PA* physical activity, *ref* reference group, *SES* socio-economic status. Model 1: adjusted for age, sex and SES; Model 2: adjusted for age, sex, SES, and comorbidities with regard to MetS risk factors at study entry. ^{}^ indicates number of N missing. Significant values (p < 0.05) are in [bold]; significant values after Bonferroni correction for multiple testing are indicated by [*]

With regard to change in sports-related PA behavior, participants who reported starting to engage in sports-related PA from < 150 min per week at baseline to ≥ 150 min per week at follow-up, had a decreased risk of incident reduced HDL (HR 0.43, 95% CI 0.25–0.74, p = 0.002) as compared to participants who remained continuously inactive. In addition, increasing sports-related PA from < 16.6 METh per week at baseline to ≥ 16.6 METh per week at follow-up, was associated with a decreased risk of incident reduced HDL (HR 0.43, 95% CI 0.24–0.77, p = 0.004), and for participants who reported a stable high sports-related PA (stable ≥ 16.6 METh per week), a decreased risk of incident reduced HDL (HR 0.55, 95% CI 0.31–0.97, p = 0.040) was found, compared to being stable inactive (Table 4).

### Association of PA with incident elevated blood pressure

Of all participants included in the analysis, 127 (42.8%) had one follow-up assessment, 67 (22.6%) participants had two follow-up assessments, 46 (15.5%) participants had three follow-up assessments, 39 (13.1%) had four follow-up assessments, and 18 (6.1%) participants had 5 follow-up assessments. The mean (SD) times of follow-up assessments was of 2.2 (1.3). After a mean follow-up of 11.4 (SD 7.5, range 5–29) years, 185 (62.3%) participants developed incident elevated BP (please refer to Fig. 1) with 3387 person-years (sum of the time spans for each participant under observation).

Habitual PA was not associated with the risk of incident elevated BP in our data set (please refer to Table 5). With regard to METh of sports-related PA, being physically active with more than 16.6 METh per week at baseline was associated with an increased risk of incident elevated BP (HR 1.49, 95% CI 1.01–2.20, p = 0.044), compared to participants who were not active.

**Table 5:** Association between PA variables and the risk of incident elevated blood pressure

No. with incident elevated BP (N = 185, 62.3%)						
	No. at risk (N = 297)	No. incident elevated BP (% of No. at risk)	Model 1 adjusted Hazard Ratio (95% CI)	p-value	Model 2 adjusted Hazard Ratio (95% CI)	p-value
Habitual PA level at baseline [min/week]						
Low (< 75)	14	8 (2.7)	ref		ref	
Medium (75–149)	74	44 (14.8)	1.23 (0.58–2.62)	0.597	1.00 (0.42–2.40)	0.995
High (≥ 150)	209	133 (44.8)	1.23 (0.59–2.54)	0.583	1.056 (0.46–2.45)	0.899
Sports-related PA level at baseline [min/week]						
Low (< 75)	162	98 (33.0)	ref		ref	
Medium (75–149)	72	45 (15.2)	1.09 (0.75–1.57)	0.652	1.11 (0.75–1.65)	0.607
High (≥ 150)	63	42 (14.1)	1.30 (0.90–1.88)	0.163	1.40 (0.94–2.09)	0.098
Sports-related intensity level at baseline [METh/week] ^{1}^						
Not intensively active (< 8.3)	169	95 (32.0)	ref		ref	
Moderately intensively active (8.3–16.5)	63	45 (15.2)	1.34 (0.93–1.92)	0.118	1.26 (0.86–1.85)	0.241
Highly intensively active (≥ 16.6)	64	45 (15.2)	1.42 (0.99–2.03)	0.058	**1.49 (1.01–2.20)**	**0.044**
Change in sports-related PA over time [min/week] ^{12}^						
Stable inactive (< 150– < 150)	171	102 (34.3)	ref		ref	
Quits activity (≥ 150– < 150)	26	20 (6.7)	**1.65 (1.01–2.69)**	**0.046**	**1.68 (1.01–2.80)**	**0.046**
Starts activity (< 150– ≥ 150)	59	38 (12.8)	0.75 (0.51–1.10)	0.142	0.69 (0.45–1.06)	0.090
Stable active (≥ 150– ≥ 150)	29	18 (6.1)	0.92 (0.55–1.53)	0.737	0.92 (0.52–1.63)	0.775
Change in sports-related intensity level over time [METh/week] ^{3}^						
Stable low (< 16.6– < 16.6)	176	103 (34.7)	ref		ref	
Decreasing (≥ 16.6– < 16.6)	30	22 (7.4)	1.47 (0.92–2.34)	0.106	1.52 (0.92–2.50)	0.101
Increasing (< 16.6– ≥ 16.6)	55	36 (12.1)	0.74 (0.50–1.10)	0.137	0.74 (0.49–1.13)	0.158
Stable high (≥ 16.6– ≥ 16.6)	33	23 (7.7)	1.03 (0.65–1.63)	0.904	1.11 (0.68–1.81)	0.668

*BP* blood pressure, *CI* confidence interval, *METh* metabolic equivalent hours, *min* minutes, *N* number of participants, *No.* Number, *PA* physical activity, *ref* reference group, *SES* socio-economic status. Model 1: adjusted for age, sex and SES; Model 2: adjusted for age, sex, SES, and comorbidities with regard to MetS risk factors at study entry. ^{}^ indicates number of N missing. Significant values (p < 0.05) are in [bold]; significant values after Bonferroni correction for multiple testing are indicated by [*]

Participants who reported cutting down sports-related PA from ≥ 150 min per week at baseline to < 150 min per week at follow-up, had an increased risk of incident elevated BP (HR 1.68, 95% CI 1.01–2.80, p = 0.046) as compared to participants who remained continuously inactive (Table 5).

### Association of PA with incident elevated blood-glucose

Of all participants included in the analysis, 283 (44.1%) had one follow-up assessment, 145 (22.6%) participants had two follow-up assessments, 108 (16.8%) participants had three follow-up assessments, 64 (10.0%) had four follow-up assessments, and 41 (6.4%) participants had 5 follow-up assessments. The mean (SD) times of follow-up assessments was of 2.1 (1.3). After a mean follow-up of 14.2 (SD 8.5, range 5–29) years, 47 (7.3%) participants developed incident elevated BG (please refer to Fig. 1) with 9082 person-years (sum of the time spans for each participant under observation).

Habitual and sports-related PA were not associated with the risk of incident elevated BG (please refer to Table 6).

**Table 6:** Association between PA variables and the risk of incident elevated blood- glucose

No. with incident elevated BG (N = 47, 7.3%)				
	No. at risk (N = 641)	No. incident elevated BG (% of No. at risk)	Model 1 adjusted Hazard Ratio (95% CI)	p-value
Habitual PA level at baseline [min/week]				
Low (< 75)	33	2 (0.3)	ref	
Medium (75–149)	143	11 (1.7)	2.12 (0.46–9.85)	0.338
High (≥ 150)	465	34 (5.3)	1.39 (0.33–5.82)	0.656
Sports-related PA level at baseline [min/week]				
Low (< 75)	345	31 (4.8)	ref	
Medium (75–149)	142	10 (1.6)	0.97 (0.46–2.06)	0.942
High (≥ 150)	154	6 (0.9)	0.54 (0.22–1.32)	0.176
Sports-related intensity level at baseline [METh/week] ^{1}^				
Not intensively active (< 8.3)	349	31 (4.8)	ref	
Moderately intensively active (8.3–16.5)	137	11 (1.7)	1.13 (0.55–2.28)	0.745
Highly intensively active (≥ 16.6)	154	5 (0.7)	0.50 (0.19–1.30)	0.154
Change in sports-related PA over time [min/week] ^{26}^				
Stable inactive (< 150 – < 150)	333	34 (5.3)	ref	
Quits activity (≥ 150 – < 150)	52	5 (0.7)	0.97 (0.38–2.50)	0.950
Starts activity (< 150 – ≥ 150)	144	6 (0.9)	0.43 (0.17–1.04)	0.062
Stable active (≥ 150 – ≥ 150)	86	1 (0.1)	**0.13 (0.02–0.92)**	**0.042**
Change in sports-related intensity level over time [METh/week] ^{6}^				
Stable low (< 16.6 – < 16.6)	348	32 (5.0)	ref	
Decreasing (≥ 16.6 – < 16.6)	54	4 (0.6)	1.03 (0.36–2.95)	0.962
Increasing (< 16.6 – ≥ 16.6)	135	10 (1.6)	0.76 (0.36–1.58)	0.457
Stable high (≥ 16.6 – ≥ 16.6)	98	1 (0.1)	**0.13 (0.02–0.99)**	**0.049**

*BG* blood-glucose, *CI* confidence interval, *METh* metabolic equivalent hours, *min* minutes, *N* number of participants, *No.* Number, *PA* physical activity, *ref* reference group, *SES* socio-economic status. Model 1: adjusted for age, sex und SES. Model 2 not reported due to small sample size of N 47 which does not allow for adjustment of more than three confounding variables. ^{}^ indicates number of N missing. Significant values (p < 0.05) are in [bold]; significant values after Bonferroni correction for multiple testing are indicated by [*]

For participants who were stable active (stable ≥ 150 min per week from baseline to follow-up), there was a decreased risk of incident elevated BG (HR 0.13, 95% CI 0.02–0.92, p = 0.042) compared to participants who were stable inactive. In addition, for participants who reported a stable high sports-related PA (stable ≥ 16.6 METh per week from baseline to follow-up), there was a decreased risk of incident elevated BG (HR 0.13, 95% CI 0.02–0.99, p = 0.049) compared to participants with a stable low sports-related PA level (Table 6).

## Discussion

The aim of this study was to examine the associations between various PA variables and new onset of five different risk factors of MetS among middle-aged males and females from a community-based sample in South-Western Germany over a period of 29 years.

Our study adds to the growing body of research on the association between PA levels and risk reduction of new onset of risk factors of MetS. Higher PA levels at baseline were associated with a decreased risk of new onset of reduced HDL, with risk reductions ranging between 37 to 42% depending on the PA variable. To our surprise, we observed a 49% increased risk of incident elevated BP in association with higher PA levels. In our data, there was no association between baseline PA levels and WC, TG or BG.

With regard to the association between changes in PA levels from baseline to follow-up, and risk of new onset of risk factors of MetS, we observed several statistically significant associations. For example, engaging in stable high sports-related PA from baseline to follow-up was associated with a a 87%-decreased risk of incident elevated BG for PA levels of 150 min or ≥ 16.6 METh per week. Furthermore, stable high levels of sports-related PA of METh from baseline to follow-up was associated with a 45%-decreased risk of incident reduced HDL. In addition, favorable changes in sports-related PA levels from baseline to follow-up, were associated with various risk reductions, ranging from a 37% decreased risk of incident elevated TG, to a 57% decreased risk of incident reduced HDL, and a 38% decreased risk for elevated WC. In contrast, reducing sports-related PA levels to below 150 min per week over time was associated with a 68% increased risk of incident elevated BP.

In general, we observed that various PA variables were associated with decreased risks of new onset of elevated TG, and reduced HDL. This is in line with previous research. For example, longitudinal studies reported that leisure time PA was associated with higher circulating levels of HDL [32], and that an higher baseline levels of PA were associated with increases in HDL in all participants, whereas decreases in TG were only found in White study participants [33]. Of note, in our study, we could show that particularly increasing PA (minutes and METh) over time seem to be beneficial to reduce the risk of new onset of elevated TG levels or reduced HDL which is also consistent with previous reports (e.g. [34]).

Several studies reported associations between PA levels and a decreased risk of incident elevated BG [35–37] or diabetes [38]. Whereas, we could not find an association between baseline PA levels but favorable change in PA over and decreased risk of incident elevated BG which is also in line with prior studies (e.g. [39, 40]). Thus, we conclude that particularly stable or increasing engagement in PA over longer periods of time is beneficial in reducing the risk of new onset of elevated BG levels.

In our data, PA does not appear to be associated with WC. In contrast, a study by Cardenas et al. reported an inverse association of leisure time PA with WC, abdominal obesity and BMI [41]. With regard to changes in PA levels over time, a large Asian cohort study reported that an increase in PA was associated with lower abdominal obesity [42]. However, a comparison between our research with other longitudinal studies is questionable, as most prior studies focused either on body weight change/obesity as outcome of interest [43], or on waist circumference as predictor variable [44], rather than outcome of interest like we did. Thus, more research on the longitudinal association between PA and risk of incident elevated WC is warranted, and researchers may also want to consider other factors such as nutrition which may play an important role on the association between PA and WC (e.g. [45]).

Also, to our surprise, our data point towards an increased risk of incident elevated BP in association with PA, particularly of higher amounts of METh. Indeed, previous studies on the association between PA levels and BP as outcome of interest have also reported conflicting results. For example, a study from Mexico reported an increased risk of hypertension in participants who were physically inactive compared to those who were highly active but found no association between total PA and hypertension [46], whereas another study found no significant association between PA and risk of hypertension [47]. Furthermore, other studies reported that higher baseline levels as well as stable PA levels are associated with lower risk of incident hypertension [37, 48, 49]. In contrast to these reports, our results showed an increased risk of elevated BP for participants who are intensively active compared to those who are inactive. However, differences between our study and previous studies may pertain to study samples and methodology, such as assessment of PA. Also, more research is needed to untangle the potential mechanisms that may underlie an association between PA and reduced or increased risk of new onset of risk factors of MetS.

Our research showed that, in addition to baseline PA, particularly PA engagement over time may be crucial to elicit potential beneficial effects on cardiometabolic health. As stated in the introduction, underlying physiological effects of PA may impact the metabolic system. In addition to its direct impact on the body, a certain lifestyle (i.e., healthy nutrition, sufficient engagement in PA) may have an impact on human body’s health in the long-term [2]. With regard to potential health benefits, PA engagement on the one hand, and stability of sports participation on the other hand appear to be important factors (i.e. [50, 51]). Additionally, future research may need to take into account quantity and quality of PA, such as type and intensity of PA over time, and preferably across the lifespan. Based on our findings, we hypothesize that maintaining or even increasing continuous engagement in sports-related PA across the lifespan may be most effective in order to achieve desirable health benefits with regard to cardiometabolic risk reduction.

A major strength of our study is the longitudinal design with a long follow-up period of 29 years. To the best of our knowledge, only few studies exist with such a long follow-up time (e.g., Harvard Alumni Study [52], Framingham Heart Study [53], National Health and Nutritional Examination Survey [54], Nurses’ Health Study [55]). In addition to previous studies, our analysis focused not only on sports-related PA at baseline, but also considered habitual activity and change in sports-related PA during follow-up. Furthermore, self-reported health status of participants was augmented by a health examination performed by a licensed physician.

Limitations of our study pertain to the rather small sample of participants with predominantly medium to high SES. Thus, our results may not be generalizable to communities of middle-aged adults with lower SES. In addition, PA was assessed by a self-reported questionnaire, which may be prone to recall bias, i.e., PA may have been over- or underestimated by participants. However, the questionnaire used in our study has been tested for reliability (test-reliability for two weeks *r* > 0.90) and internal consistency (α = 0.94) [24]. Furthermore, we did not examine changes in PA between childhood or youth and adulthood, or between early and middle adulthood. It is known that participation in PA varies across the lifespan, e.g., sports behavior is rather stable from childhood to youth [56], whereas PA behavior in adulthood is instable [57]. Therefore, an objective assessment of PA behavior (i.e., using accelerometry) would add beneficial information about PA and should be considered in future research. Furthermore, our results are not adjusted for multiple comparison, and this may lead to potential risk of bias. Therefore, a post-hoc Bonferroni correction can be applied to adjust p-values for multiple testing according to the formula (adjusted p-value = 0.05/number of tests) [58]. Thus, the adjusted p-value after Bonferroni Correction is 0.01 (= 0.05/5), for each outcome of interest respectively. Results that remained significant after correction are indicated by * in the result tables (please refer to Tables 2 and 6). This applies to the associations between changing PA (increasing PA from below to more than 150 min/16.6 METh per week) and incident reduced HDL and elevated TG. In addition, with regard to change in PA behavior as predictor variable in our analyses, we only considered the first and latest information on PA available in our datasets. Thus, potential fluctuations in PA behavior between other measurements are not reflected which may have led to biased findings. Therefore, further analyses should consider other statistical analyses to better account for fluctuations over time such as latent growth curve analyses.

In our study, we considered the first occurrence of onset of risk factors of MetS as the outcome of interest. However, by definition, the status of these five risk factors may fluctuate over time in participants, particularly as we had several measurement points. As this sample selection definition may led to potential bias, future studies should thus not only focus on the first event of incident risk factor of MetS in a dataset, but also consider change in status of risk factors of MetS. Due to the focus on risk factors of MetS (i.e., WC, HDL, TG, BP or BG) it is possible to predict incident MetS [59]. Furthermore, we did not examine the association between risk factors and their dependence within each other. Thus, in addition to calculating HRs, future studies may explore a different statistical approach, e.g. linear-mixed modelling. In addition, in our analysis, we only examined the potential impact of PA on MetS risk factors. However, MetS is multifaceted and incidence of MetS risk factors, as well as PA pattern, may be influenced by other behavioral and/ or lifestyle-related variables such as nutrition [14, 60–63] or fitness [22, 64]. Finally, even though we conducted a longitudinal study to examine the association between PA (which was considered the predictor) and incident outcome of interests, we cannot answer the question of cause and effect, and inverse causality may thus be possible. Therefore, more research is needed to untangle the longitudinal associations between PA and incident risk factors of MetS, and our study also needs to be confirmed by prospective studies conducted in other communities.

## Conclusions

In conclusion, our data shows that engagement in PA, particularly at medium or high levels, as well as starting PA engagement, or maintaining and increasing PA levels over time are associated with decreased risk of new onset of MetS risk factors, particularly elevated TG, reduced HDL, elevated WC, and elevated BG. These findings have implications for PA promotion in middle-aged adults aimed at increasing metabolic health, and underline the importance of structured PA health promotion programs and public health strategies, even at community-based levels.

## Data Availability

The datasets generated and analyzed for the current study are not publicly available due to the strict ethical standards as required by the ethics committee of the Karlsruhe Institute of Technology, Germany. However, data may be available from the corresponding author on reasonable request.

## Abbreviations

BG: Blood-glucose
BP: Blood pressure
CI: Confidence interval
HDL: High-density lipoprotein cholesterols
HR: Hazard Ratio
M: Mean
METh: Metabolic equivalent-hours
MetS: Metabolic syndrome
PA: Physical activity
SD: Standard deviation
SES: Socio-economic status
TG: Triglycerides
WC: Waist circumference
WHO: World Health Organization
